# Research progress of B7-H3 in malignant tumors

**DOI:** 10.3389/fimmu.2025.1586759

**Published:** 2025-05-30

**Authors:** Shuaixiang Zhao, He Zhang, Guanning Shang

**Affiliations:** ^1^ Department of Bone and Soft Tissue Oncology, Department of Orthopaedic, Shengjing Hospital, China Medical University, Shenyang, Liaoning, China; ^2^ The First Clinical College, Liaoning University of Traditional Chinese Medicine, Shenyang, Liaoning, China

**Keywords:** B7-H3(CD276), malignant tumor, targeted therapy, immunity, drug trials

## Abstract

B7 homolog 3 (B7-H3, also known as CD276) is a novel member of the B7 immune protein family. There is a marked difference in the expression and distribution of B7-H3 protein and mRNA between normal and tumor tissues, with widespread expression in tumor tissues and a close relationship with tumor progression. B7-H3 activates or inhibits tumor immune responses by binding to receptors on the surface of immune cells. Apart from participating in tumor immune activities, it has regulatory effects on non-immunological functions, such as tumor migration and invasion, angiogenesis, glycometabolism, and drug resistance. Thus, it has important biological functions in regulating the progression of malignant tumors. Current research on the structure, function, and therapeutic methods of B7-H3 is continuously breaking new ground, deepening our understanding of B7-H3, and promoting the development of therapeutic drugs targeting this new protein. This review briefly discusses the structure and distribution of B7-H3, as well as its immune and non-immune functions in the progression of cancer. It also summarizes the research progress on drugs targeting B7-H3 and the latest developments in clinical trials, highlighting their significant potential for the treatment of malignant tumors.

## 1 Introduction

The treatment and prognosis of malignant tumors have always been challenging in the medical field. The current mainstream treatment still fundamentally relies on resection of the lesion, but this does not prevent the metastasis and recurrence of cancer. Immune checkpoint blockade is a recently developed strategy for cancer treatment. It activates the body’s antitumor ability through immune recognition of antigens and enhances the immune system to suppress or even eliminate tumors (1, 2).

B7-H3 is a newly identified immune checkpoint protein, which has been found to be highly expressed in various types of cancerous tissues and is closely associated with tumor progression (3). It is currently known that there are two isoforms of the human transmembrane B7-H3 protein: 2Ig-B7-H3 and 4Ig-B7-H3. Current studies indicate that 4Ig-B7-H3 is the predominant expression form of B7-H3 in the human body and is associated with immune evasion mechanisms in the tumor microenvironment. There is currently no evidence to suggest that 2Ig-B7-H3 is involved in tumor immunity in humans (4–6). Current research findings indicate that B7-H3 exhibits a significant stimulatory effect on immune cells and is associated with tumor proliferation, migration, invasion, apoptotic mechanisms, angiogenesis, glycometabolism, drug resistance, and radioresistance (7). Over the two decades since the initial discovery of B7-H3, research on this molecule has been relentless, and our understanding of B7-H3 has deepened progressively. In recent years, research on the mechanisms of B7-H3 has advanced significantly. In this article, we provide a concise overview of the immunological and non-immunological roles of B7-H3 in various types of tumors and summarize the global development progress of drug therapies targeting B7-H3.

## 2 Biology of B7-H3

### 2.1 B7 family

B7 family members are a class of transmembrane proteins that bind to different T-cell or B-lymphocyte surface receptors and generate immune responses of immune cells through co-stimulation or co-inhibition (8, 9). The basic structural characteristics of B7 family members are associated with extracellular immunoglobulin variable-like (IgV) and immunoglobulin constant-like (IgC) domains. Their IgV and IgC domains, as well as the leader sequence, transmembrane, and cytoplasmic domains, are all encoded by exons (9). Currently known B7 family members include B7-1 (CD80), B7-2 (CD86), B7-H1 (PD-L1), B7-DC (PD-L2), B7-H2 (ICOSLG), B7-H3 (CD276), B7-H4 (VTCN1), B7-H5 (VISTA), B7-H6 (NCR3LG1), and B7-H7 (HHLA2), and they possess approximately 20% amino acid sequence identity among them (10, 11) (**Table 1**).

**Table 1:** Members, receptors, structures and distributions of the B7 Family.

Member	Receptor	Structure	Ligand-receptor interaction	Distribution
B7-1	CD28, CTLA-4	IgV-IgC	Co-stimulation or co-inhibition	Immune cells
B7-2	CD28, CTLA-4	IgV-IgC	Co-stimulation or co-inhibition	Immune cells
B7-H1	PD-1	IgV-IgC	Co-stimulation or co-inhibition	Immune cells, Endothelial cells, Normal tissues, Tumors
B7-DC	PD-1, RGMb	IgV-IgC	Co-stimulation or co-inhibition	Immune cells
B7-H2	ICOS	IgV-IgC	Co-stimulation	Immune cells, Fibroblasts, Endothelial and epithelial cells
B7-H3	TLT-2	IgV-IgC or IgV-IgC-IgV-IgC	Co-stimulation or co-inhibition	Immune cells, Bone marrow, Tumors
B7-H4	VTCN1	IgV-IgC	Co-stimulation or co-inhibition	Immune cells, Tumors
B7-H5	KIR3DL3,TMIGD2	IgV-IgC-IgV	Co-inhibition	Immune cells, Tumors
B7-H6	NKp30	IgV-IgC	NK cell: co-stimulation	Immune cells, Tumors, Placenta
B7-H7	KIR3DL3,TMIGD2	IgV-IgC-IgV	Co-stimulation or co-inhibition	Immune cells, Tumors, Endothelial cells, Epithelial cells

### 2.2 Structure of B7-H3

Chapoval et al. identified a B7 family molecule in a cDNA-derived library from human dendritic cells (DCs), which was an independent reverse transcription polymerase chain reaction (RT-PCR) product of the human tumor cell line THP-1 and the DC library. This molecule encodes a 316 amino acid protein, and its extracellular receptor-binding domain shares 20%–27% identity with previously discovered B7 family members (B7-1, B7-2, B7-H1, and B7-H2). Hence, it was named B7-H3 (12). In subsequent studies, human B7-H3 was encoded on chromosome 15 (4), and mouse B7-H3 was encoded on chromosome 9 (13). There is 80% identity and 93% similarity between human B7-H3 and mouse B7-H3 (mB7-H3) (13). B7-H3 exists in two forms in biological organisms: transmembrane protein and soluble protein forms (sB7-H3) (12, 14).

The basic structure of B7-H3 consists of a pair of extracellular immunoglobulin variable-like (IgV) and immunoglobulin constant-like (IgC) domains (IgV-IgC), a transmembrane region, and a highly diversified short cytoplasmic tail containing 45 amino acids, with a signal peptide at its amino (-NH2) terminus (12). Peter et al. cloned human B7-H3 and found slight differences in its encoding compared with previously discovered B7-H3 encoding. The deduced structure of the cloned B7-H3 protein consists of a short leader sequence, two pairs of IgV and IgC domains (IgV-IgC-IgV-IgC), a transmembrane domain, and a short cytoplasmic tail. However, the two pairs of Ig-like domains of human B7-H3 showed a high identity of 95%. Thus, it is identified as 4Ig-B7-H3 (B7-H3b), a subtype of B7-H3 commonly found in humans (4, 13). sB7-H3 was first identified by Zhang, who demonstrated through enzyme-linked immunosorbent assay (ELISA) that sB7-H3 can be detected in cell lines expressing the transmembrane protein B7-H3. He also confirmed that sB7-H3 is cleaved from the surface of activated T cells, monocytes, and monocyte-derived dendritic cells (MDDCs) by matrix metalloproteinases (MMPs) and can bind to the B7-H3 receptor on T cells (14). Chen’s study discovered a new isoform of B7-H3, resulting from selective splicing of the fourth intron of B7-H3, and named it spliced sB7-H3. This isoform exerts a negative regulatory effect on T cells, inhibiting T cell proliferation. Additionally, it was found that sB7-H3 is closely related to the prognosis of patients with liver cancer (15).

**Figure 1:**
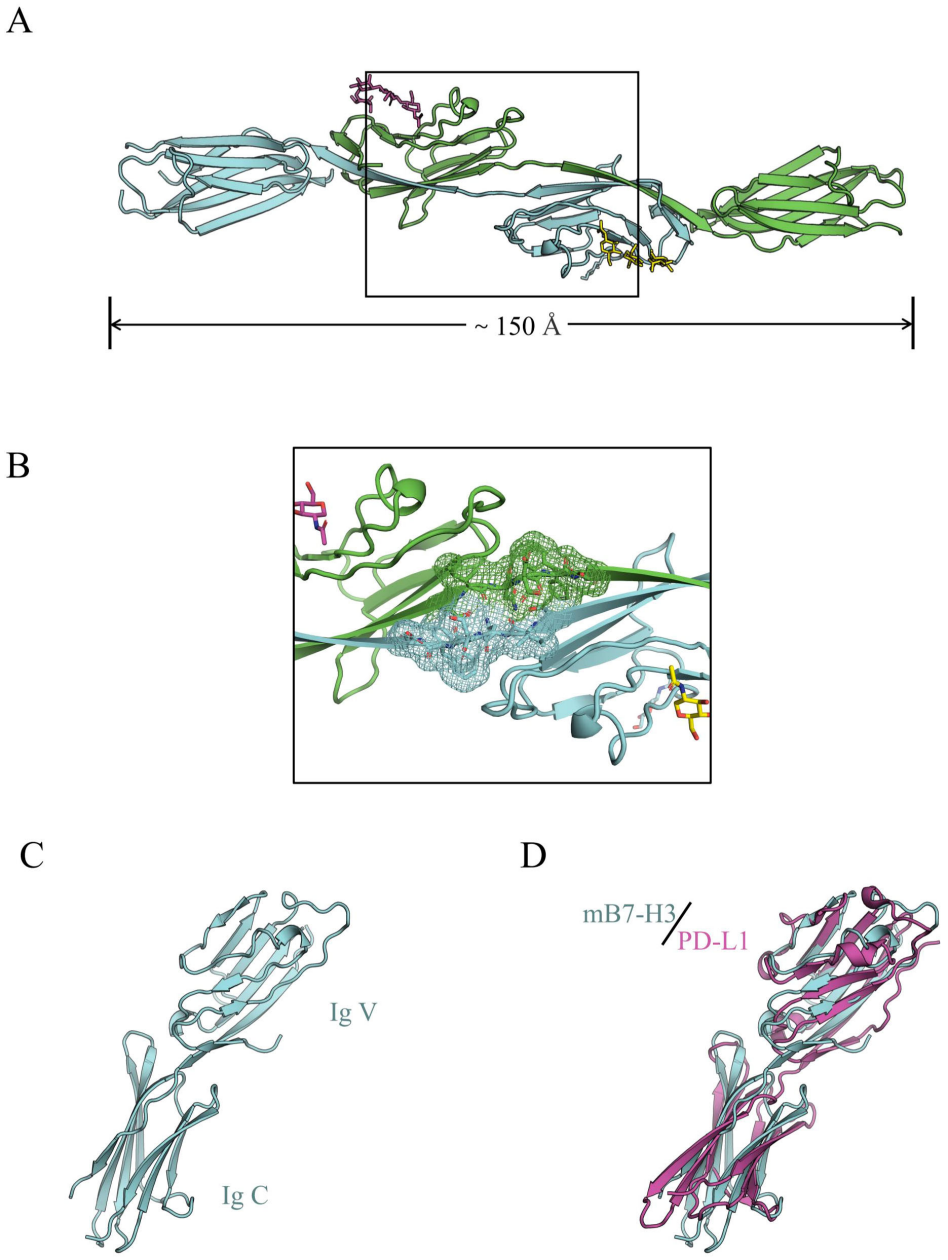
**(A)** The dimeric form of mB7-H3, with gray and cyan representing two different subunits; **(B)** The electron density of the FG loop bridging sequence in mB7-H3; **(C)** The monomeric structural model of mB7-H3; **(D)** The structural model of mB7-H3 compared with the human PD-L1 structural model (16).

Vigdorovich et al. reported that the crystal of mB7-H3 exhibited diffraction consistent with the space group P6122 (a = 100.9 Å, b = 100.9 Å, c = 188.2 Å, α = 90°, β = 90°, γ = 120°, wavelength: 0.9789 Å, resolution: 50.96–2.97 Å) (**Figure 1**) (16).Sun found that 2Ig-B7-H3 in mice can participate in immune functions both as a transmembrane protein and as sB7-H3, while 4Ig-B7-H3 can only engage in immune activities by binding to different receptors (17). Furthermore, the study found that the connecting segment between the F chain and G chain of two adjacent IgV-like domains in mB7-H3 does not adopt the classic IgV-like domain FG loop conformation but rather is composed of G chains exchanged between two adjacent IgV-like domains. Therefore, the crystallization of mB7-H3 is an unusual dimeric form (18).

### 2.3 Distribution and expression of B7-H3

**Figure 2:**
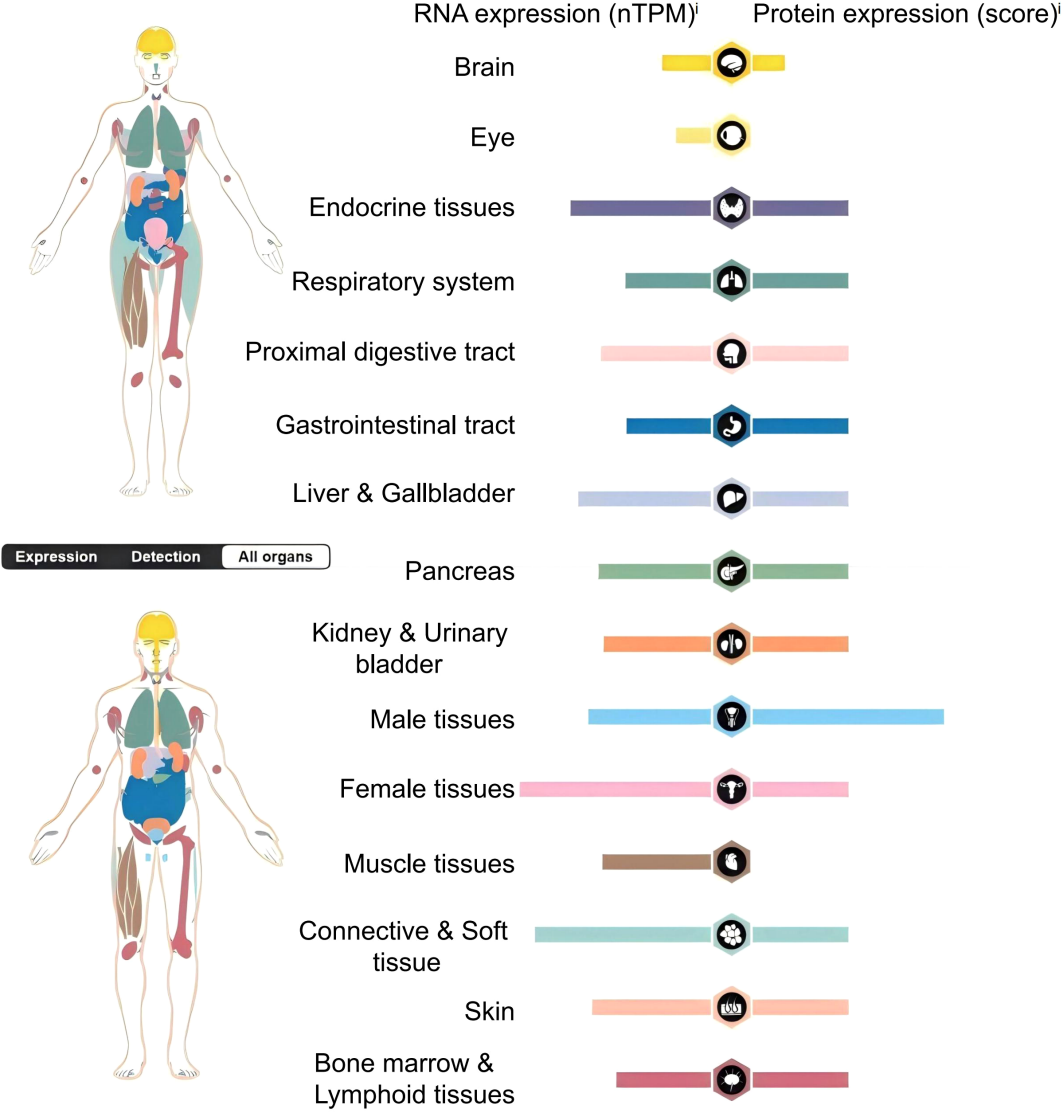
Distribution and expression of B7-H3 in the human body.nTPM (normalized Transcripts Per Million) is a standardized method used to quantify gene expression levels, which eliminates the effects of sequencing depth and gene length differences, thereby allowing for more accurate comparisons of expression data between different samples.

Studies have confirmed that B7-H3 mRNA is widely observed in both normal human tissues and tumor cells, with lower expression in normal tissues and widespread expression in cancer cells (18–20). According to the Human Protein Atlas, the RNA of B7-H3 is ubiquitously and widely expressed in normal human tissues and organs. However, the protein expression of B7-H3 is absent in the human eye and muscle tissues, and its expression in other tissues and organs is significantly lower than the RNA levels of B7-H3 (**Figure 2**). B7-H3 protein can be found on non-immune cells, such as fibroblasts and osteoblasts, but its expression on immune cells, such as natural killer (NKs) cells, DCs, and monocytes, requires induction (18, 19).

B7-H3 protein expression levels are regulated by various factors, among which the post-transcriptional regulatory mechanism of microRNA (miRNA) is relatively representative. Studies have shown that numerous distinct miRNAs affect the expression of B7-H3 protein (21, 22). MiRNAs are small RNAs composed of more than 20 endogenous nucleotides. They have multiple functions, including regulating the developmental timing, cellular spatial architecture, and physiological functions of cells and tissues (23). To verify the effect of miRNAs on B7-H3 protein expression in breast cancer, Nygren selected 20 different miRNAs, and the study found that 13 of them (miR-214, miR-363*, miR-326, miR-940, miR-29c, miR-665, miR-34b*, miR-708, miR-601, miR-124a, miR-380-5p, miR-885-3p, and miR-593) can directly bind to the 3′-UTR of B7-H3 (24). Gao found that both miR-214 and B7-H3 are expressed in multiple myeloma (MM), and that miR-214 can directly target the 3′ UTR of B7-H3 to influence its expression (25). Similarly, Wang et al.’s research found that miR-124 can directly regulate the expression of B7-H3 protein in osteosarcoma cells (26). Zhou found that TGF-β1 in colorectal cancer cells can promote B7-H3 protein expression by upregulating miR-155 and inhibiting miR-143 expression, indicating that the expression of B7-H3 in colorectal cancer cells is regulated by TGF-β1 (27). Li et al. discovered that in human head and neck squamous cell carcinoma, the 3′-untranslated region (3′-UTR) of B7 homolog 3 (B7-H3) can bind to microRNA-214-3p (miR-214-3p), and overexpression of miR-214-3p inhibits the expression of B7-H3 (28). MiR-29a, miR-29b, and miR-29c have been demonstrated to be associated with B7 homolog 3 (B7-H3) expression in studies (29–31). Anup found that the miR-29 family (miR-29a, miR-29b, miR-29c) targets B7-H3 in neuroblastoma (NB) cells, promoting NK cell activation and NK-mediated cytotoxicity, thereby inducing an anti-tumor immune response through the interaction of miR-29a, miR-29b, and miR-29c with mRNA in NB cells (32). Meng discovered that miR-29c directly inhibits B7-H3 expression in ovarian cancer, while also stimulating NK cell activation to suppress tumor progression (31).

Besides post-transcriptional regulation by miRNAs, the expression of B7-H3 is also influenced by other protein and mRNA modifications. Protein methylation is a complex post-translational modification mechanism, where lysine methyltransferases (KMTs) or protein arginine methyltransferases (PRMTs) use S-adenosyl-L-methionine (SAM) as a methyl donor to act on proteins, thus occurring on lysine or arginine residues (33). Ubiquitination is also a common post-translational modification that affects the malignant biological behaviors of tumor cells (34). Meng found that protein arginine methyltransferase 5 (PRMT5) promotes B7-H3 expression by mediating the m6A modification of its mRNA via meR316-ALKBH5, impacting colorectal cancer (CRC) prognosis (35). The ubiquitin-conjugating enzyme E2T (UBE2T) is a protein that acts on protein ubiquitination in cells. Shi discovered that in the study of the mechanism of brain metastasis in triple-negative breast cancer (TNBC), UBE2T promotes B7-H3 expression by directly binding to and ubiquitinating CDC24 (Cell Division Cycle 42), a GTPase that aids in cell structure remodeling, cell migration, cell cycle regulation, and signal transduction, thereby influencing breast cancer progression (36). Histone lactylation is a mechanism of histone metabolic reprogramming that can regulate tumor progression, with lactate being essential in this process. Ma discovered in their study that lactate influences the expression of B7-H3 in tumor cells through histone lactylation, inhibiting the function of CD8+ T cells and promoting tumor immune evasion (37).

In addition to miRNA regulation, the overexpression of immunoglobulin-like transcript 4 (ILT4) in non-small cell lung cancer (NSCLC) cells activates the phosphoinositide 3-kinase (PI3K)/protein kinase B (AKT)/mammalian target of rapamycin (mTOR) signaling pathway, leading to increased expression of B7-H3 protein (38). Ets-like protein-1 (ELK-1) is a transcription factor involved in gene expression (39), and in lung adenocarcinoma (LUAD), ELK-1 promotes B7-H3 protein expression by binding to the promoter region of B7-H3 (40). SUPT20H (SP20H) is a transcription factor that regulates protein expression. Experimental research has found that SP20H downregulation can activate the p38/MAPK-eIF4E signaling pathway, thereby stimulating the upregulation of B7-H3 protein expression (41).

## 3 Function of B7-H3

### 3.1 Immune functions of B7-H3

B7-H3 protein may be involved in the regulation of T cell-mediated immune responses. Initially, B7-H3 was considered a co-stimulatory molecule that could be utilized to influence T-cell activation and IFN-γ production. IFN-γ is primarily an antitumor immune factor produced by NK cells and activated T cells (42). Chapoval et al. stimulated purified T cells with human CD3 monoclonal antibody (mAb) in a B7-H3 context and demonstrated that B7-H3 can co-stimulate the growth of CD3+ and CD4+ T cells and enhance the generation of CD8+ T cells *in vitro*. Additionally, B7-H3 selectively promotes the production of IFN-γ (12). Hashiguchi et al. identified a B7-H3 receptor, which is a Triggering Receptor Expressed on Myeloid Cells (TREM)-like transcript-2 (TLT-2, a member of the immunoglobulin superfamily) expressed on myeloid cells. The study demonstrated that B7-H3 can positively stimulate CD8+ T cells to promote T-cell activation. B7-H3 engages in positive co-stimulation of T cells by binding to TLT-2 expressed on CD4+ and CD8+ T cells, enhancing T-cell activation and thereby augmenting tumor immunity (43). Loos et al. conducted an immunohistochemical analysis of B7-H3 expression and its correlation with T-cell counts in 68 cases of pancreatic carcinoma tissue. The results indicated a significant association between B7-H3 expression and the number of CD8+ T cells (P = 0.018). Moreover, high B7-H3 expression was significantly correlated with IFN-γ upregulation (P = 0.0225) (44). Luo et al. injected P815 tumor cells into mice and observed that B7-H3 can directly stimulate T cells in the absence of antigen-presenting cells, thereby enhancing the immunological function of CD8+ T cells within the tumor (45). In gastric cancer cells, B7-H3 expression is negatively correlated with CD8+ T-cell concentration (46).

B7-H3, in addition to its co-stimulatory effect on T cells, possesses co-inhibitory functions. Interleukin-2 (IL-2) drives the clonal expansion of T cells. Hence, the production of IL-2 is a hallmark of T-cell activation. Prasad et al., in their study on the co-stimulatory role of murine B7-H3, found that it inhibits IL-2 production by downregulating the NF-κB, NFAT, and AP-1 signaling pathways, thereby inhibiting T-cell activation (47). Chen et al., in their study on the immunologic function of porcine B7-H3, found through a controlled experiment that IFN-γ and IL-2 production was significantly inhibited in the presence of 4Ig-B7-H3-Fc (48). Vigdorovich et al., in their study on the activity of B7-H3, also found that B7-H3 has an inhibitory effect on T-cell activation (16).

B7-H3 inhibits NK cell cytotoxicity. Castriconi et al., through their research on the impact of 4Ig-B7-H3 on NK cells, found that the degree of lysis of CHO-K1 cells transfected with 4Ig-B7-H3 by NK cells was significantly lower than that of non-transfected CHO-K1 cells, indicating that the 4Ig-B7-H3 molecule protects neuroblastoma from NK cell attack by inhibiting NK cell cytotoxicity (49).

**Figure 3:**
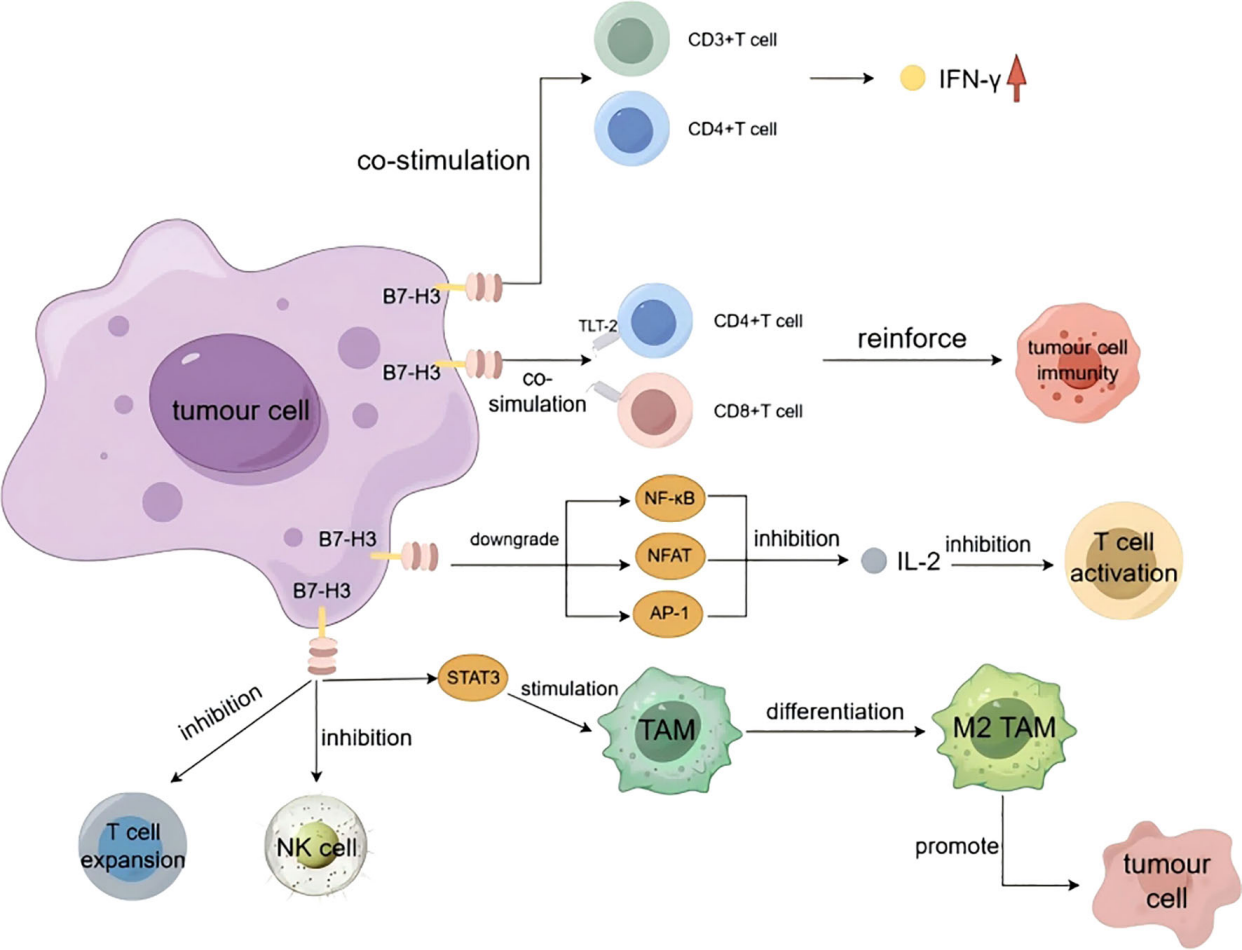
The immune functions of B7-H3.

B7-H3 influences tumor immunity by modulating the differentiation of tumor-associated macrophages (TAMs). TAMs primarily originate from the differentiation of circulating monocytes, impacting tumorigenesis, progression, and metastasis. They can suppress CD8+ T-cell responses, thereby promoting tumor development (50–54). TAMs are primarily divided into two subtypes: M1 and M2. M1-like TAMs can directly mediate cytotoxicity or exert antitumor effects through antibody-dependent cell-mediated cytotoxicity (ADCC), while M2-like TAMs can promote tumor cell proliferation, migration, and progression (55). Current research indicates that hepatocellular carcinoma tissue influences the B7-H3/Stat3 signaling pathway by releasing inflammatory stimuli, thereby inducing the polarization of M2-type TAMs (56). In patients with colorectal carcinoma, B7-H3 promotes the differentiation of M2-type TAMs by activating receptors on the surface of TAMs (57). Furthermore, in high-grade serous ovarian cancer tissues, B7-H3 is involved in the immunosuppressive activity mediated by the CCL2-CCR2-M2 macrophage axis (58) (**Figure 3**).

### 3.2 Non-immune functions of B7-H3

B7-H3, identified as a novel immune checkpoint protein, not only influences the growth and metastasis of cancer cells by participating in immune responses but also impacts tumor progression through various non-immune pathways. These include sustaining proliferative signaling, evading growth suppressor factors, resisting cell death, achieving replicative immortality, inducing/facilitating angiogenesis, activating invasion and metastasis, and reprogramming cellular metabolism.

#### 3.2.1 Regulation of tumor proliferation, migration, and invasion

The proliferation, invasion, and migration of neoplastic cells are hallmarks of cancer progression (59). Invasion is the first step in tumor metastasis. The dissemination that occurs during the metastatic process can lead to severe and acute organ failure, making tumor metastasis a major cause of cancer-related mortality (60). C-X-C chemokine receptor type 4 (CXCR4) is a receptor for stromal cell-derived factor-1 (SDF-1) and is closely associated with cancer cell migration and invasion. In a study, Li et al. discovered that B7-H3 activates the AKT, ERK, and Jak2/Stat3 signaling pathways by affecting CXCR4, thereby inducing cancer cell invasion and migration (61, 62). Chen et al. found that siRNA-B7H3 downregulation led to decreased migration and invasion rates of FEMX-I melanoma (16% and 30%, respectively), decreased migration of FEMX-V melanoma cells (26%) and decreased migration and invasion rates of MA11 breast cancer cells (both 45%). This study demonstrated that B7-H3 can inhibit the migration and invasion of tumor cells (63). Research has found that major vault protein (MVP) regulates the transport of tumor suppressor miRNA, thereby promoting tumor migration (64). Liu et al. have demonstrated that B7-H3 can stimulate the MVP/mitogen-activated protein kinase (MEK) signaling axis to promote cancer cell metastasis (65). In pancreatic cancer, B7-H3 has been found to promote tumor invasion and metastasis through the TLR4/NF-κB pathway (66). In NSCLC, B7-H3 promotes epithelial-mesenchymal transition (EMT) via the PI3K/AKT pathway (67). B7-H3 promotes EMT in hepatocellular carcinoma through the Jak2/Stat3/Slug signaling pathway (68). In colorectal cancer tissues, B7-H3 induces cancer cell migration and invasion through the Jak2/Stat3/MMP-9 signaling pathway (69). In studies on cancer stem cells of human head and neck squamous cell carcinoma, B7-H3 has been found to activate the AP-1 pathway, leading to cell invasion and metastasis (70). Furthermore, in studies on cervical cancer, ovarian cancer (OV), pancreatic cancer, glioma, pancreatic ductal adenocarcinoma, and clear cell renal cell carcinoma, B7-H3 has been identified as a target associated with the promotion of cancer cell proliferation, invasion, and metastasis (71–78).

#### 3.2.2 Regulation of apoptosis

Apoptosis is a genetically controlled, autonomous cell death mechanism. Resistance to apoptotic mechanisms can lead to the continuous development of malignant tumor cells and ultimately exacerbate the degree of malignancy (79). Previous studies found that the JAK2/STAT3 pathway activates the apoptosis inhibitors survivin, Mcl-1, Bcl-2, and Bcl-XL to block the caspase cascade and the initiation of apoptotic mechanisms in tumor cells (80). Zhang et al. reported that B7-H3 overexpression leads to elevated levels of phosphorylated Jak2 and Stat3. The expression of the anti-apoptotic proteins, Bcl-2 and Bcl-XL, decreased with the reduction in phosphorylation levels of Jak2 and Stat3, while the expression of pro-apoptotic protein Bax correspondingly increased (81). Previous reports have indicated that low concentrations of doxorubicin (DOX) can induce apoptosis (82). In a study investigating the function of B7-H3 in low-dose DOX-induced senescence in colorectal cancer cells, Wang et al. found that B7-H3 knockdown promotes DOX-induced apoptosis, where high expression of B7-H3 activates the AKT/TM4SF1/SIRT1 signaling pathway, exacerbating the resistance to low-dose DOX-induced apoptosis (83). Han et al. discovered that in cervical cancer tissues, B7-H3 may regulate the expression of apoptosis-related proteins, including PARP-1, Caspase-8, Bax, Bcl-2, and Bcl-XL, through the E7/Rb signaling pathway, thereby affecting cellular apoptosis (84). Furthermore, evidence of enhanced cell resistance to apoptosis due to overexpression of B7-H3 has been observed in gastric and breast cancers (85, 86).

#### 3.2.3 Impact on tumor angiogenesis

Tumor growth and metastasis require nutrients, which are contingent upon tumor angiogenesis, one of the hallmarks of cancer (87). Vascular endothelial growth factor (VEGF) is an angiogenic factor and one of the primary proteins associated with vasculogenesis. It regulates vascular permeability, endothelial cell proliferation, and the recruitment of circulating endothelial progenitor cells. VEGF can directly act on cancer cells, influencing their invasiveness (88). Sun et al.’s study on breast cancer tissues demonstrated a significant correlation between B7-H3 and VEGF expression, with B7-H3 downregulation leading to increased VEGF protein levels (89). Wang et al. discovered that overexpression of B7-H3 in colorectal cancer cells significantly increased VEGF concentration. They activated AKT, NF-κB, and STAT3 with B7-H3 and observed that the VEGF expression level was higher in the NF-κB group than in the other two groups. Ultimately, they determined that B7-H3 could promote VEGF expression by activating the NF-κB signaling pathway, thereby promoting the formation of blood vessels in colorectal cancer (90). Similarly, Wu et al. discovered that B7-H3 influences angiogenesis in colorectal cancer tissues through the AKT1/mTOR/VEGFA pathway (91). Xie et al., in their investigation of the impact of B7-H3 expression in pancreatic cancer cells, demonstrated that B7-H3 facilitates VEGF secretion through the TLR4/NF-κB pathway (66). A study on the impact of B7-H3 expression on clear cell renal carcinoma found that B7-H3 promotes tumor angiogenesis through the Tie-2 pathway (92). Furthermore, evidence from studies on intrahepatic cholangiocarcinoma showed that B7-H3 influences tumor angiogenesis (93).

#### 3.2.4 Regulation of cancer cell metabolism

The Warburg effect is a metabolic pattern specific to tumor cells. Compared to normal cells, tumor cells consume extremely high amounts of glucose to achieve proliferation. Moreover, cancer cells do not completely oxidize glucose but instead convert a large portion of it into lactate (94). Lim et al. showed that B7-H3 overexpression can promote glycolysis in cancer cells and the Warburg effect in breast cancer cells (95). Previous studies have indicated that hypoxia-inducible factor-1α (HIF-1α) plays a role in regulating cancer cell glycolysis (96). Reactive oxygen species (ROS) can maintain the stability of HIF-1α function. Lim et al. compared cells with B7-H3 knockdown to control cells and found decreased HIF-1α levels in the B7-H3 knockdown group. Ultimately, the experimental results showed that B7-H3 can regulate cancer cell glycometabolism by mediating HIF-1α stability through ROS (95). Similarly, Li et al. discovered that B7-H3 modulates the expression of HIF-1α in oral squamous cell carcinoma, thereby affecting cancer cell glycolysis through the activation of the PI3K/AKT/mTOR signaling pathway (72). Alpha-enolase (ENO1) has been shown to facilitate the Warburg effect in cancer cells (97). Zuo et al., utilizing a lactate and ATP detection kit, observed a significant decrease in ATP and lactate production in HeLa/pshB7-H3 and HeLa/anti-B7-H3 cells compared to other control groups. These findings suggest that silencing or inhibiting B7-H3 impedes glycolysis in HeLa cells (98). B7-H3 modulates glycometabolism in neuroblastoma through the Stat3/c-Met pathway, thereby promoting tumor cell migration and invasion (99). Furthermore, studies in esophageal squamous cell carcinoma and colorectal cancer have demonstrated that B7-H3 can modulate tumor cell metabolism (100, 101).

#### 3.2.5 Regulation of tumor drug resistance and radioresistance

**Figure 4:**
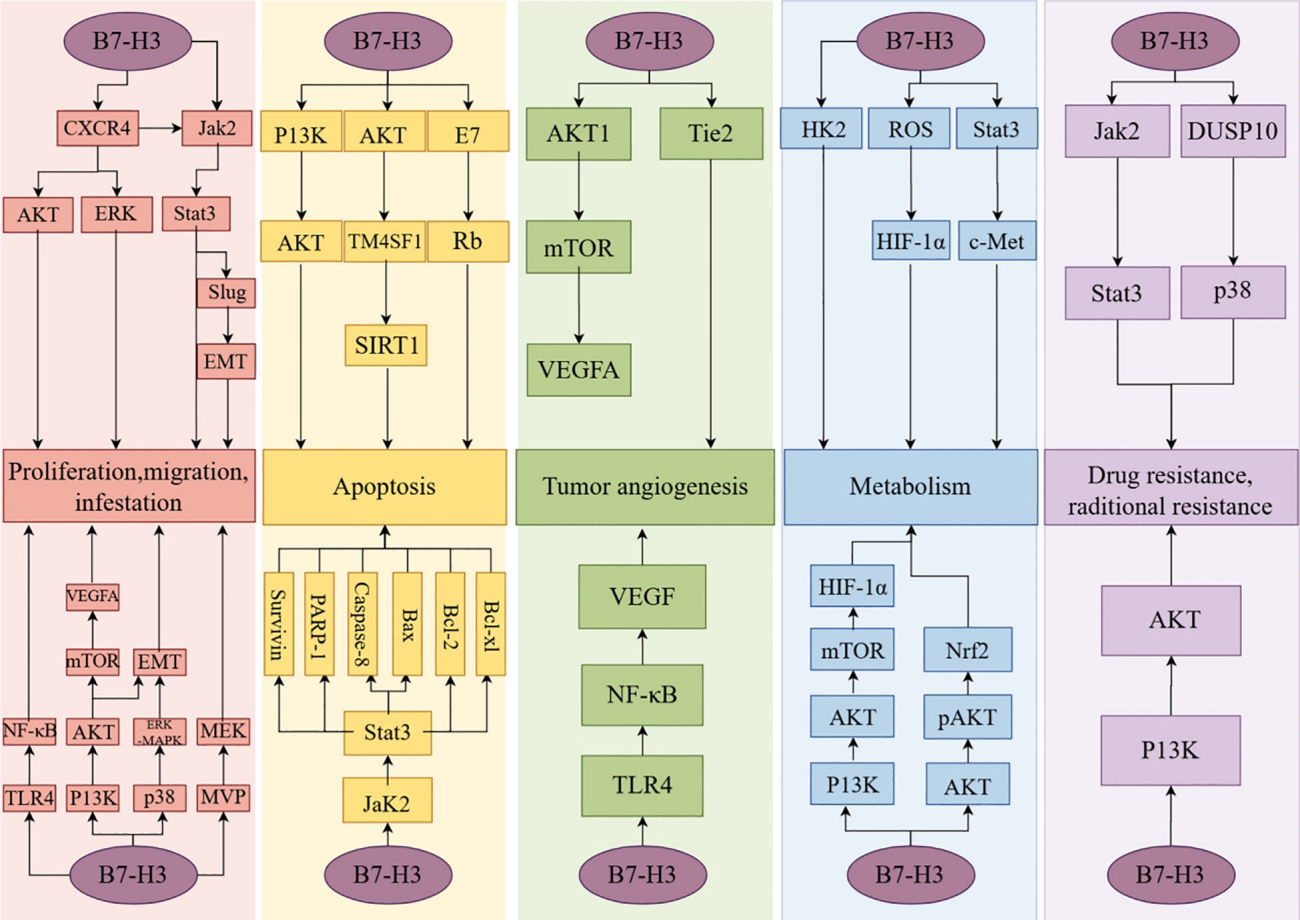
The non-immune functions of B7-H3.

Chemotherapy and radiotherapy are commonly used in the treatment of malignant tumors. Although these two therapeutic modalities are well established, their long-term use can lead to increased tumor drug resistance and radioresistance and may accelerate cancer progression, posing significant challenges for the treatment and prognosis of malignant tumors. Early studies indicate that glycolysis in cancer cells is associated with chemoresistance, and proteases that regulate glycolysis are also related to drug resistance (102). Shi et al. established a mouse model of colorectal cancer with B7-H3 overexpression, and experimental data indicated that B7-H3-induced glycolysis enhancement confers drug resistance in colorectal cancer cells (101). Liu et al. discovered that B7-H3 enhances chemoresistance in breast cancer cells by activating the Jak2/Stat3 signaling pathway, thereby reducing the sensitivity of breast cancer cells to paclitaxel (85). Zhou et al. reported that overexpression of B7-H3 can activate the PI3K/AKT signaling pathway, conferring drug resistance and promoting tumor growth (103) (**Figure 4**).

Additionally, Suh et al. found that B7-H3 is highly expressed during the late stages of osteoblast differentiation in mouse embryonic development. B7-H3 promotes osteoblast differentiation and osteogenesis during the later stages of osteogenesis (104). Furthermore, research findings indicate that B7-H3 expression levels in adipose progenitor cells of mice and humans can modulate the metabolism of these cells, thereby affecting the degree of obesity in mice or humans (105).

## 4 Research progress on the role of B7-H3 in cancer

### 4.1 Research progress of B7-H3 in breast cancer

Joshi et al. assessed the role of B7-H3 in breast cancer subtypes and brain metastasis of breast cancer. They discovered a significant positive correlation between B7-H3 expression and high expression of the tumor proliferation marker Ki67. B7-H3 protein expression is positively correlated with high tumor-infiltrating lymphocytes (TILs) in breast cancer. Moreover, B7-H3 protein expression was detected in 90% of cases of brain metastasis of breast cancer, suggesting that high expression of B7-H3 can promote breast cancer cell proliferation and brain metastasis of breast cancer (106). By analyzing 1,082 breast cancer specimens selected from the TCGA database, Cheng et al. found that B7-H3 protein expression was higher in triple-negative/basal-like breast cancer (TNBC) tissue samples compared to other subtypes of breast cancer, suggesting a close association between B7-H3 expression and the malignancy of TNBC (107). Mei et al. discovered that the expression of B7-H3 in TNBC tissue samples correlates with collagen content and the degree of immune cell infiltration. They classified tumors into four types based on their immune and collagen characteristics. Tumors with high TIL levels and no collagen deposition were defined as “Hot & Non-armored” tumors, while those with low TIL levels and collagen deposition were termed “Cold & Armored” tumors. The remaining two types were sequentially defined as “Hot & Armored” and “Cold & Non-armored” tumors. Experimental research revealed that B7-H3 is highly expressed in “Cold & Armored” tumors and is closely associated with collagen deposition levels (108) (**Table 2**).

**Table 2:** Functions and mechanisms of B7-H3 in cancer.

Cancer types	Function	Mechanism	Reference
Breast cancer	Proliferation, migration and invasion	Raf/MEK/ERK signaling pathway, MVP/MEK signaling pathway	(65, 152)
	Drug resistance	Jak2/Stat3 signaling pathway	(85)
	Metabolism	Mediate HIF1α through ROS	(95)
	Anti-apoptosis	Jak2/Stat3 signaling pathway	(85)
	Angiogenesis	Inhibit the secretion of VEGF	(89)
Prostate cancer	Promote tumor growth	High expression of B7-H3 promotes PTEN/TP53-deficient prostate cancer	(111)
	Anti-immunity	Inhibit the T cells and NK cells	(109)
	Migration	B7-H3 siRNA inhibits fibronectin adhesion and cancer cell metastasis	(113)
Gastric cancer	Increase the stemness of cancer cells	AKT/pAKT/Nrf2 signaling pathway	(120)
	Anti-apoptosis	PI3K/AKT signaling pathway	(86)
	Anti-immunity	Inhibit the immunity of T cells	(46)
Lung cancer	Proliferation, migration and invasion	PI3K/AKT signaling pathway	(67)
	Anti-apoptosis	Promote the expression of HIF-1α by upregulating the p - NF-κB	(128)
Melanoma	Drug resistance	MAPK or AKT/mTOR signaling pathway, DUSP10-p38 signaling axis	(135, 136)
	Promote tumor growth	Jak2/Stat3 signaling pathway	(134)
AML	Affecting prognosis	Not mention	(138–140)
Brain tumor	Affecting prognosis	Not mention	(141–145)

### 4.2 Research progress on the role of B7-H3 in prostate cancer

Prostate cancer is a common type of cancer in men. Treatment options for prostate cancer have matured, but the significant challenge of poor prognosis persists. Thus, immune-targeted therapy may emerge as a promising alternative (109). An analysis of 2,111 prostate cancer patient tissue samples revealed that the B7-H3 gene is within the top 19th percentile of genes expressed in prostate cancer. Compared to primary prostate cancer, the expression of B7-H3 is significantly increased in metastatic castration-resistant prostate cancer. Additionally, the study found a correlation between B7-H3 and the androgen receptor, with the androgen receptor inhibiting the expression of B7-H3 (110). In immunogenetic analysis of PTEN/TP53-deficient prostate cancer, B7-H3 was identified as the most prominent immune gene. It was observed that high expression of B7-H3 promotes tumor growth and suppresses the immune functions of T cells and NK cells in PTEN/TP53-deficient prostate cancer (111). Guo et al. investigated the expression of B7-H3 protein during the progression of metastatic prostate cancer to castration-resistant prostate cancer, determining that the expression of B7-H3 is negatively correlated with DNA repair genes and positively correlated with the expression of ETS-related gene (ERG) in prostate cancer. Concurrently, although there is a loss of B7-H3 protein in prostate cancers with neuroendocrine differentiation phenotype, B7-H3 is expressed in the vast majority of prostate cancer patients, including those with neuroendocrine characteristics (112). Yuan et al. discovered in their experiments investigating the role of B7-H3 in prostate cancer tissues that B7-H3 siRNA inhibits fibronectin adhesion in PC-3 cells, thereby preventing cancer cell metastasis (113). Yong et al. developed an exosome (EVs)-based liquid biopsy detection method to assess B7-H3 expression in tissue samples from patients with metastatic castration-resistant prostate cancer (mCRPC). This method not only reflects the temporal dynamics of B7-H3 expression but also offers more treatment options for patients, indicating that B7-H3 is a biomarker and potential therapeutic target for prostate cancer (114).Furthermore, numerous studies have indicated that B7-H3 is a novel and promising therapeutic target for prostate cancer (109, 115, 116).

### 4.3 Research progress on the role of B7-H3 in gastric cancer

Gastric cancer is one of the most common causes of cancer-related mortality worldwide, with the majority of cases originating from Asia, and the prognosis for patients with advanced gastric cancer is particularly poor (117, 118). Wu et al. analyzed 102 gastric cancer tissue samples and found that B7-H3 protein expression in gastric cancer tissues was not associated with patient age, sex, lymph node metastasis, tumor location, size, or depth, but was correlated with gastric cancer patient prognosis and survival time (119). Tumor stem cells are one of the factors influencing tumor therapy. Through their investigation into the mechanism by which B7-H3 affects the stemness of gastric cancer cells, Xia et al. discovered that B7-H3 modulates the metabolism of glutathione to increase the stemness of gastric cancer cells via the AKT/pAKT/Nrf2 signaling pathway (120). Li et al. discovered that granulocyte-macrophage colony-stimulating factor is initially produced in gastric cancer cells, activating the Jak2/Stat3 signaling pathway to mediate the activation of tumor-associated neutrophils and the expression of B7-H3 (121). Currently, for patients with early gastric cancer, endoscopic resection and lymph node dissection are commonly employed treatments, while for those with advanced gastric cancer, immune protein-targeted therapy is utilized. However, the number of targets is currently limited (122). The emergence of the novel target B7-H3 may improve the treatment outcomes and prognosis of gastric cancer (123–125).

### 4.4 Research progress on the role of B7-H3 in lung cancer

Lung cancer is generally classified into two types: small cell lung cancer (SCLC) and NSCLC. Sun et al.’s study of 38 patients with squamous cell carcinoma showed no correlation between B7-H3 expression and patient age, sex, smoking history, or tumor differentiation. In the pathological tissue samples of 56 patients with lymph node metastasis, 21 showed overexpression of B7-H3. B7-H3 overexpression was significantly associated with lymph node metastasis in NSCLC (126). By detecting the expressions of PD-L1, B7-H3 and B7-H4 in the tissue samples of SCLC cells, the results showed that, overall, the expression level of B7-H3 was higher than that of PD-L1 and B7-H4 (127). Recent studies have discovered that the expression level of B7-H3 in lung cancer cells is positively correlated with the number of monocytes/macrophages. B7-H3 promotes the expression of HIF-1α by upregulating the phosphorylation levels of NF-κB, enhancing the anti-apoptotic ability of monocytes/macrophages, and facilitating their aggregation in the tumor microenvironment (128). A study examining the relationship between B7-H3 and lung adenocarcinoma found that the overall survival curve of the high-expression group of B7-H3 was significantly lower than that of the low-expression group, indicating an association between B7-H3 and the prognosis of lung adenocarcinoma. B7-H3 can promote the progression of EMT by regulating molecules related to EMT, thereby facilitating the proliferation and metastasis of lung adenocarcinoma (129). In another study focusing on NSCLC, B7-H3 protein overexpression was significantly associated with poor prognosis and reduced survival rates (130).

### 4.5 Research progress on the role of B7-H3 in melanoma

Melanoma, a malignant neoplasm arising from melanocytes, is triggered by ultraviolet radiation and is a rare yet highly lethal disease, accounting for 75% of skin cancer-related deaths (131, 132). Previous studies have indicated that the expression of phosphorylated STAT3 (p-STAT3) in melanoma cells is associated with poor prognosis (133). Our experiments demonstrated that B7-H3 modulates the expression of phosphorylated signal transducer and activator of transcription 3 (p-STAT3) and cyclin D1 through the JAK2/STAT pathway. The quantity of B7-H3 mRNA is significantly increased in melanoma compared with normal skin, benign nevi melanocytes, and moles, and continues to rise with the progression of cancer stages. The upregulation and downregulation of B7-H3 expression significantly affect the migration and invasion of melanoma cells (134). Studies have found that knockdown of B7-H3 in metastatic melanoma tissue increases the sensitivity of melanoma cells to chemotherapy with dacarbazine (DTIC), binimetinib (MEK inhibitor), everolimus (mTOR inhibitor), and triciribine (AKT inhibitor) (135). B7-H3 enhances chemoresistance in tumors by downregulating DUSP10, which in turn mediates p38 MAPK activation (136). These studies have elucidated the close association between B7-H3 and the growth and prognosis of melanoma and have demonstrated the potential of B7-H3 as a novel therapeutic target.

### 4.6 Research progress on the role of B7-H3 in AML

Acute myeloid leukemia (AML) is a malignancy of the human hematopoietic system that disrupts normal hematopoietic function, ultimately leading to bone marrow failure and death (137). Sylwia’s study on AML analyzed data from 77 patient samples and found that B7-H3 expression is associated with the overall survival (OS) of AML patients. Patients with low B7-H3 expression exhibited significantly better prognoses than those with high B7-H3 expression; thus, Sylwia proposed that B7-H3 warrants further investigation as a prognostic marker for AML (138). Anudishi discovered that inhibiting B7-H3 expression in AML patient samples enhances NK cell-mediated apoptosis in AML cells, thereby promoting AML cell death and extending OS in AML patients (139). Sylwia also demonstrated that an optimized targeted B7-H3 immunotherapy drug was effective in treating 68 AML patient samples, with flow cytometry analysis showing that the optimized anti-B7-H3 drug induces NK cell activation and AML cell death by inhibiting B7-H3 expression on AML cells (140). These studies indicate that B7-H3 has the potential to serve as a prognostic marker for AML.

### 4.7 Research progress on the role of B7-H3 in brain tumors

Gliomas are the most common malignant brain tumors, and current treatment methods include surgery, radiotherapy, and chemotherapy; however, the treatment outcomes remain suboptimal. Wang found a significant correlation between isocitrate dehydrogenase (IDH) mutations, which are indicative of early glioma progression, and B7-H3 expression in high-grade glioma cells. He observed that higher B7-H3 expression is associated with poorer prognosis in glioma patients (141). Glioblastoma (GBM), a brain tumor originating from the central nervous system (CNS), is primarily treated with surgery, radiation therapy, and various regimens of temozolomide; however, the average survival time for GBM remains short, with a five-year survival rate of only 6.8% (142). Marina analyzed brain tissue samples from GBM patients and non-cancerous brain tissue samples, finding that B7-H3 expression was 26.1% higher in GBM samples compared to non-cancerous samples. Furthermore, B7-H3 expression was significantly elevated in GBM tissues relative to normal tissues, with studies indicating a negative correlation between B7-H3 levels and overall survival (OS) in GBM patients (143). Ramazan conducted a follow-up study on 86 IDH wild-type (wt) GBM patients and found that those with high B7-H3 expression exhibited lower OS, confirming B7-H3 as a significant predictor of OS in these patients (144). Proctor reported that B7-H3 expression in cancer cells was nearly 100% in most tested meningioma specimens, making it the most highly expressed immune checkpoint protein (145). Existing studies have established B7-H3 as a prognostic biomarker for gliomas, significantly correlating with OS in glioma patients.

### 4.8 Research progress of B7-H3 in other cancers

Zang et al., in their investigation of ovarian cancer, observed that the expression of B7-H4 was 100%, while that of B7-H3 was 93%. They also found that elevated B7-H3 expression level was associated with increased cancer recurrence and mortality (146). Proctor et al. found that in the majority of tested meningioma specimens, B7-H3 expression in cancer cells approached 100%, being the most highly expressed immune checkpoint protein (145). Yamato et al. initially demonstrated that B7-H3 expression is significantly higher in human pancreatic cancer tissues than in normal pancreatic tissues and suggested that B7-H3 plays a crucial role in the treatment and prognosis of pancreatic cancer (147). Yuko discovered that B7-H3 is associated with the glycolytic pathway in epithelioid mesothelioma cells and promotes ATP production within the cells. Furthermore, he found that high expression of B7-H3 mRNA is closely related to poor prognosis in patients with epithelioid mesothelioma, proposing that B7-H3 could serve as a key biomarker for this condition (148). Furthermore, B7-H3 protein expression has been demonstrated to play a significant role in cancer migration, invasion, treatment, and prognosis in esophageal carcinoma (149), endometrial carcinoma (150), and renal cell carcinoma (151).

## 5 Research progress on targeted B7-H3 therapies

Immunotherapy is an effective clinical treatment for advanced and refractory cancers. Several studies have confirmed that B7-H3 can serve as a novel tumor immune marker, and several drugs targeting B7-H3 have entered preclinical or trial phases. Although the receptor for B7-H3 is currently unknown, it is anticipated that once the receptor for B7-H3 is identified, the development of drugs targeting this emerging target will accelerate significantly.

### 5.1 Monoclonal antibody

MAbs are a class of antibodies produced by B lymphocytes that target a single specific antigen. They can be categorized into two types: non-conjugated and conjugated mAbs. These antibodies can directly bind to pathogens, immediately disrupting the invasion pathways of pathogens and preventing their entry into cells, or they can activate the immune response by binding to the Fc domain on the surface of the antibody and the Fcγ receptors (FCGRs) on various immune cells (153, 154). Enoblituzumab (also known as MGA-271) is a B7-H3-targeting, Fc-engineered mAb that enhances ADCC in B7-H3-expressing tumor cell lines. In bladder cancer xenografts, MGA-271 inhibited cancer cell growth, and a similar reduction in tumor recurrence was observed in renal cell carcinoma xenografts. Additionally, no significant adverse reactions were noted after administering doses of MGA-271 as high as 150 mg/kg to cynomolgus monkeys (155). 8H9 is an mAb with a broad targeting profile generated by the fusion of mouse myeloma SP2/0 cells with spleen lymphocytes from BALB/c mice immunized against human neuroblastoma. Immunohistochemical assays have revealed its high reactivity with brain tumors, sarcomas, and neuroblastomas (156). Ahmed et al. demonstrated through computational modeling that the 8H9 ligand binds to the FG loop of B7-H3, thereby inhibiting the expression of B7-H3 (157). Wu et al. developed a novel mAb, 24F-Hu-mut2, targeting human B7-H3 to treat esophageal squamous cell carcinoma. This mAb binds to the IgC1 and IgC2 domains of B7-H3, influencing B7-H3 protein expression and subsequently affecting tumor growth (158) (**Table 3**).

**Table 3:** Summary of clinical trial progress and mechanisms of B7-H3-Targeting drugs (https://www.clinicaltrials.gov/).

Drug	Type	Mechanism	Cancer types	Trial number
Enoblituzumab (MGA271)	mAb	Utilizing cytotoxic effects to inhibit tumor growth.	Prostate cancer	NCT02923180
IBI-334	BsAb	Blocking the signal transduction of EGFR and B7-H3 to inhibit tumor growth.	Unresectable, locally advanced or metastatic solid tumors	NCT05774873
TAK-280	BsAb	Activating CD3+ T cells to inhibit tumor progression.	Unresectable, locally advanced or metastatic cancers	NCT05220098
HS-20093	ADC	Specifically binding to B7-H3 to inhibit tumor growth.	Advanced solid tumors	NCT05276609
		Specifically binding to B7-H3 to inhibit tumor growth.	Relapsed or refractory osteosarcoma and other sarcomas	NCT05830123
Ifinatamab Deruxtecan (DS-7300a, I-DXd)	ADC	Releasing DXd to inhibit the activity of TOPI and induce apoptosis of cancer cells.	Advanced or malignant solid tumors	NCT04145622
MHB088C	ADC	Releasing a more potent TOPI inhibitor to inhibit the activity of TOPI and induce apoptosis of cancer cells.	Advanced or metastatic solid tumors	NCT05652855
TX103	CAR-T	Stimulating T cells to inhibit tumor growth.	Recurrent glioblastoma	NCT05241392
SCRI-CARB7H3(s)	CAR-T	T cells lentivirally transduced to express a B7H3-specific CAR and EGFRt.	DIPG,DMG, and recurrent or refractory CNS tumors.	NCT04185038
4-1BBζ B7H3-EGFRt-DHFR	CAR-T	T cells extracted from blood and genetically modified to express B7H3-specific receptors	Relapsed or refractory non-CNS solid tumors	NCT04483778
ILB-3101	ADC	ILB-3101 can specifically bind to B7-H3 and play a role in inhibiting tumor growth.	Advanced solid tumors	NCT06426680
Obrindatamab (MGD009/MGA012)	Combination therapy	MGA012 inhibits T cell checkpoint and MGD009 exerts cytotoxicity.	Relapsed or refractory advanced solid tumors	NCT03406949

### 5.2 Bispecific antibody

Bispecific antibodies (BsAbs) are artificially synthesized antibodies that can bind to two different antigens. They are designed to kill tumor cells by directing the binding to target antigens and antigens on effector cells (159). IBI-334 is a BsAb developed for targeted therapy against B7-H3 and EGFR. It has demonstrated a more potent ADCC effect in xenograft models of lung cancer, bronchioalveolar carcinoma, and lymph node metastasis of pulmonary mucoepidermoid carcinoma than wild-type antibodies. Furthermore, when administered at a dosage of 120 mg/kg/week, IBI-334 exhibited only mild skin hardening at the injection site in cynomolgus monkeys, with no significant adverse reactions observed (160). TAK-280 is a newly developed Conditional Bispecific Redirected Activation (COBRA) T-cell engager currently under investigation in clinical trials to treat metastatic castration-resistant prostate cancer and NSCLC, with results yet to be disclosed. Upon internalization into tumor cells, TAK-280 initially binds to B7-H3 and, subsequently, within the protease-rich tumor microenvironment, engages with CD3ϵ. The dimerization of TAK-280 with CD3ϵ leads to CD3+ T-cell activation, triggering a cytolytic antitumor response targeting cells co-expressing B7-H3 (161).

### 5.3 Antibody-drug conjugate

An antibody-drug conjugate (ADC) consists of an mAb, a cytotoxic payload, and a chemical linker. Upon entering the human body, the mAb on the surface of the ADC binds to the target antigen on the tumor cell surface. After recognition, the ADC is internalized into the tumor cell, where the cytotoxic payload is released, leading to the death of the tumor cell by binding to the specific target antigen (162). HS-20093, a fully humanized IgG1 ADC, was evaluated in two clinical trials involving patients with advanced-stage solid tumors and those with relapsed or refractory osteosarcoma. Both trials showed that HS-20093 exhibits marked antitumor activity, possesses a reliable safety profile, and has acceptable toxicity (163, 164). DNA topoisomerase I (TOP1) is overexpressed in various cancers and plays a role in promoting DNA replication and cell division, thereby stimulating tumor growth (165). Ifinatamab Deruxtecan (DS-7300a) is an effective ADC targeting B7-H3, composed of a humanized anti-B7-H3 IgG1 mAb (MABX-9001a), a Top I inhibitor payload known as DXd, and a cleavable tetrapeptide linker. Upon internalization by cancer cells, it releases DXd, which inhibits Top I activity, leading to apoptosis in cancer cells. DS-7300a has demonstrated potent antitumor activity and reliable safety in preclinical mouse and monkey patient-derived xenograft (PDX) models (166). DS-7300a, a DNA Top I inhibitor-based antibody-drug conjugate targeting B7-H3, exerts potent antitumor activities in preclinical models, including PDX models, against a variety of pediatric solid tumors expressing B7-H3 (167). MHB088C is a novel ADC targeting B7-H3, comprising an activated mAb against B7-H3 and a potent Top I inhibitor, which exhibits 5 to 10 times greater efficacy than the DXd payload. In preclinical drug trials, MHB088C demonstrated 3–10 times stronger antitumor activity and a more favorable safety profile than DS-7300a. In the initial phase of human clinical trials, MHB088C provided significant therapeutic benefits for patients with SCLC, with one patient experiencing an 80% reduction in tumor volume (168).

### 5.4 Chimeric antigen receptor T-cell therapy

Chimeric antigen receptor T (CAR-T) cell therapy is an innovative and precision-targeted cancer treatment approach known for its high efficacy and durable antitumor response. CAR is a synthetic receptor engineered to redirect immune cells to specifically recognize antigens, consisting of four main components: the extracellular target antigen-binding domain, a linking segment, a transmembrane domain, and one or more intracellular signaling domains (169). Majzner et al. developed a B7-H3-targeting CAR-T cell therapy based on the high B7-H3 expression in pediatric solid tumors. This therapy was engineered from the well-characterized mAb MGA271, which has been optimized for human use and exhibits potent antitumor activity in various pediatric solid tumor PDX models, including osteosarcoma, Ewing’s sarcoma, and medulloblastoma (170). Tang et al. conducted the first-in-human trial of B7-H3-targeting CAR-T cell therapy. CAR targeting B7-H3 consisted of a human CD8α leader peptide, an anti-B7-H3 single-chain variable fragment (scFv), a human CD8α hinge region, a human CD8α transmembrane domain, a 4-1BB/CD3ζ intracellular signaling domain, and a truncated CD19 (CD19t) for CAR detection. The subject was a patient with anaplastic meningioma. The clinical study results indicated that this CAR-T cell therapy could suppress tumor cell activity without severe adverse effects (171). Navin’s team conducted a clinical trial targeting patients aged 0–26 with relapsed or refractory non-central nervous system (non-CNS) solid tumors, genetically modifying T cells from the subjects’ blood to target B7-H3. The results demonstrated that these modified cells elicited a significant anti-tumor response in the subjects without notable cytotoxicity (172). Nicholas conducted a clinical trial involving repeated intraventricular (ICV) administration of B7-H3-specific autologous CAR T cells, transduced with a lentivirus, for adults under 26 years with relapsed or refractory CNS tumors and children over one year with diffuse intrinsic pontine glioma (DIPG). The results indicated that the treatment regimen using B7-H3-targeted CAR T cells (SCRI-CARB7H3(s)) was feasible for CNS tumor patients aged 1–26 years, with subjects tolerating doses of CAR T cells ranging from 1×10^7 to 10×10^7 cells. However, the clinical efficacy of this trial could not be accurately assessed, related to factors such as the number of participants and the age of the subjects (173).

### 5.5 Other drug immunotherapies

In addition to the four commonly mentioned immunotherapies, other immunotherapeutic strategies should also be considered, such as Bispecific Killer Cell Engagers or Trispecific Killer Cell Engagers, which consist of two or three single-chain variable fragments, single-domain antibodies, and antigen-binding fragments. The design of small molecule inhibitors targeting the B7-H3 protein’s IgV domain, based on the structural characteristics of the FG loop, allows drugs to target B7-H3 directly. Additionally, combination therapies involving multiple immune checkpoints should be explored, among other strategies (16, 174–176).

## 6 Conclusion and future perspectives

B7-H3 has been proven to be a member of the B7 immune protein family. Owing to its overexpression in the majority of cancer tissues and its ability to regulate T-cell activation, B7-H3 is involved in tumor growth, development, and prognosis, making it a potential new target. Further investigation into the mechanisms underlying the generation of soluble B7-H3 and its specific functions within the tumor microenvironment will enhance our understanding of its dual role in tumor immune regulation. Additionally, simultaneously assessing the synergistic or competitive effects of soluble B7-H3 and transmembrane B7-H3 in targeted therapies will provide critical evidence for optimizing treatment strategies.

To date, numerous drugs targeting B7-H3 have been developed, with some demonstrating efficacy and safety in clinical trials. However, the receptors of B7-H3 and the specific mechanisms of action of B7-H3 in the human body remain unclear, significantly hindering drug development. Further research into the receptors of B7-H3 and elucidation of its mechanisms of action in normal tissues and other diseases beyond cancer are needed. This will enable the design and development of more effective and safer therapeutic drugs, offering hope for definitive resolution of the high recurrence and mortality rates associated with malignant tumors.
