# Correction: Research progress of B7-H3 in malignant tumors

**DOI:** 10.3389/fimmu.2025.1641733

**Published:** 2025-06-17

**Authors:** Shuaixiang Zhao, He Zhang, Guanning Shang

**Affiliations:** ^1^ Department of Bone and Soft Tissue Oncology, Department of Orthopaedic, Shengjing Hospital, China Medical University, Shenyang, Liaoning, China; ^2^ The First Clinical College, Liaoning University of Traditional Chinese Medicine, Shenyang, Liaoning, China

**Keywords:** B7-H3(CD276), malignant tumor, targeted therapy, immunity, drug trials

In the published article, there was an error in the Affiliation(s). Instead of “1The First Clinical College, Liaoning University of Traditional Chinese Medicine, Shenyang, Liaoning, China,2Department of Bone and Soft Tissue Oncology, Department of Orthopaedic, Shengjing Hospital, China Medical University, Shenyang, Liaoning, China”, it should be “1Department of Bone and Soft Tissue Oncology, Department of Orthopaedic, Shengjing Hospital, China Medical University, Shenyang, Liaoning, China,2The First Clinical College, Liaoning University of Traditional Chinese Medicine, Shenyang, Liaoning, China”.

The authors apologize for this error and state that this does not change the scientific conclusions of the article in any way. The original article has been updated.

